# Supplementary material for: Impacts of predator-mediated interactions along a climatic gradient on the population dynamics of an alpine bird
Source: Proc Biol Sci. 2020 Dec 23;287(1941):20202653. doi: 10.1098/rspb.2020.2653 (PMC7779518; doi:10.1098/rspb.2020.2653)
Supplement: Supplementary material [file rspb20202653supp1.docx]

Supplementary material for:

# Impacts of predator-mediated interactions along a climatic gradient on the population dynamics of an alpine bird

Diana E. Bowler, Mikkel A. J. Kvasnes, Hans C. Pedersen, Brett K. Sandercock, Erlend B. Nilsen

DOI: 10.1098/rspb.2020.2653

Questions to: [diana.e.bowler@gmail.com](mailto:diana.e.bowler@gmail.com)

Content:

- Additional figures and tables (Pages 2-6)
- Appendix A – Effect of harvesting (Page 7)
- Appendix B – Effect of rodents (Pages 8-12)
- Appendix C – BUGS code for hierarchical Bayesian model (Pages 13-22)


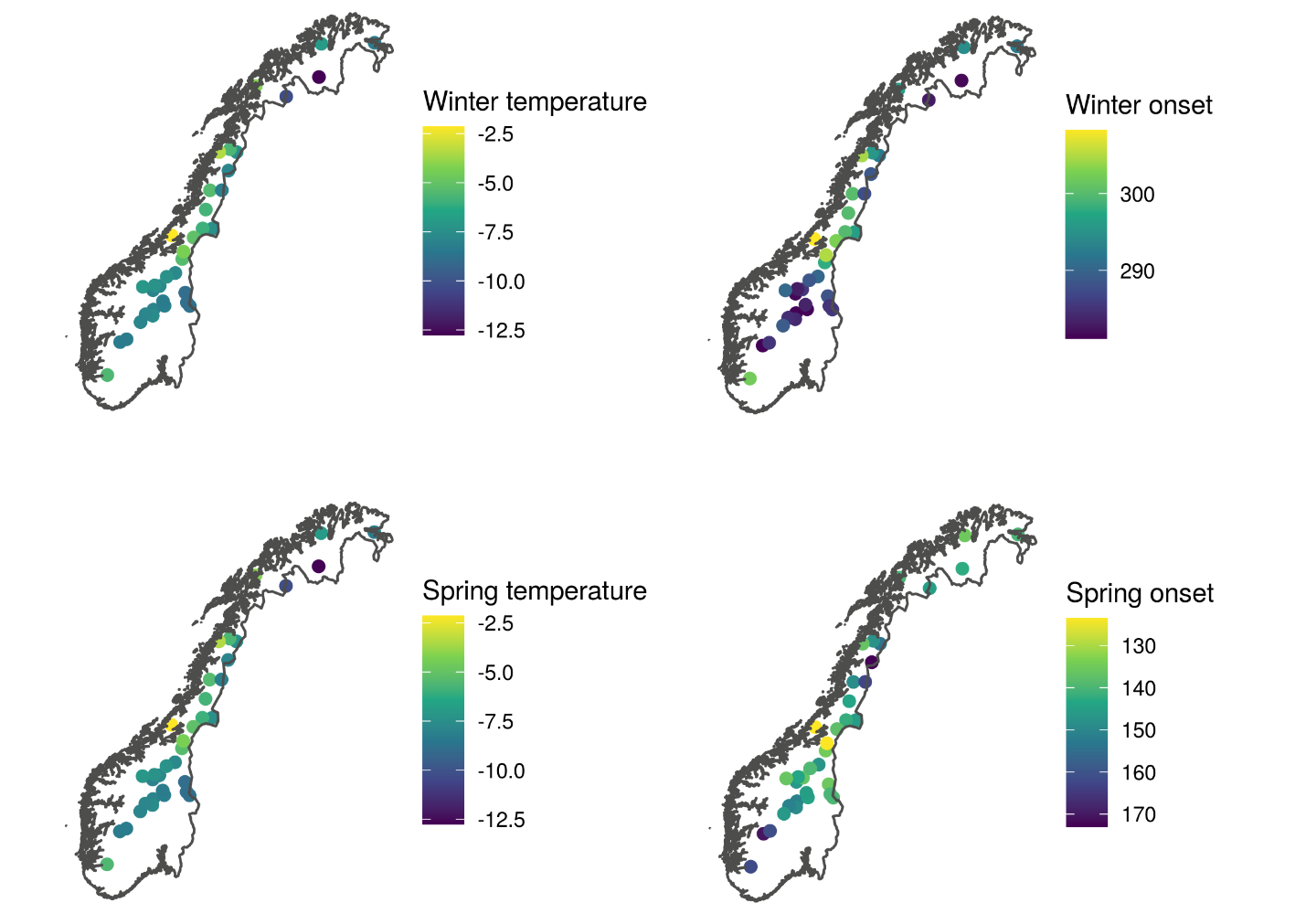


**Fig. S1** The location of each of the 36 survey regions (a survey region is a cluster of neighbouring transects), shaded by the mean value of each climatic variable during 2007–2017.


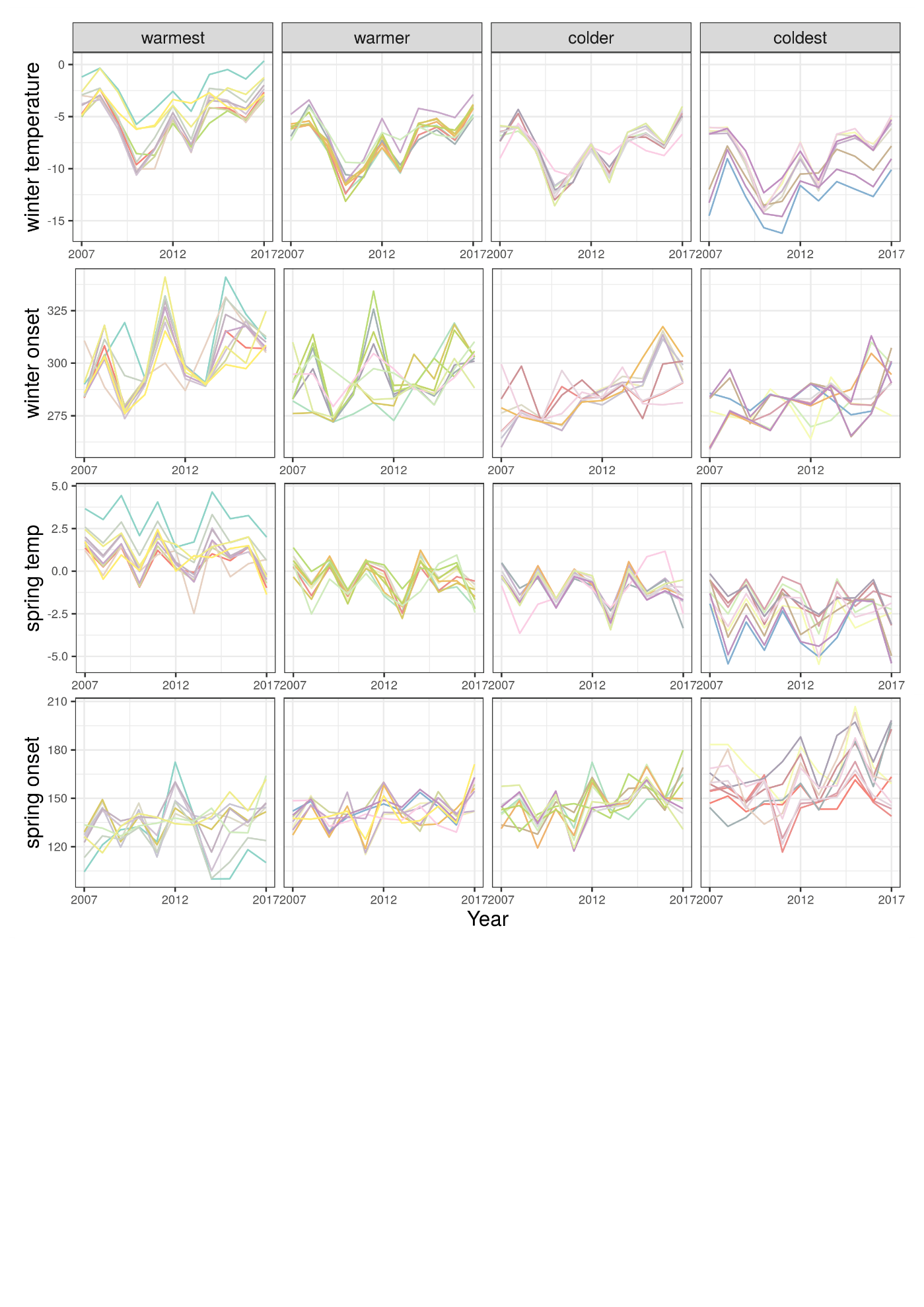


**Fig. S2** Time-series of the climatic variables between 2007 and 2017. Each line is the mean value for line transects within each survey region. Temperature values are in °C. Onset values are year day values. See main text for more details. Panels are split by quartiles of each of the respective climatic variables.


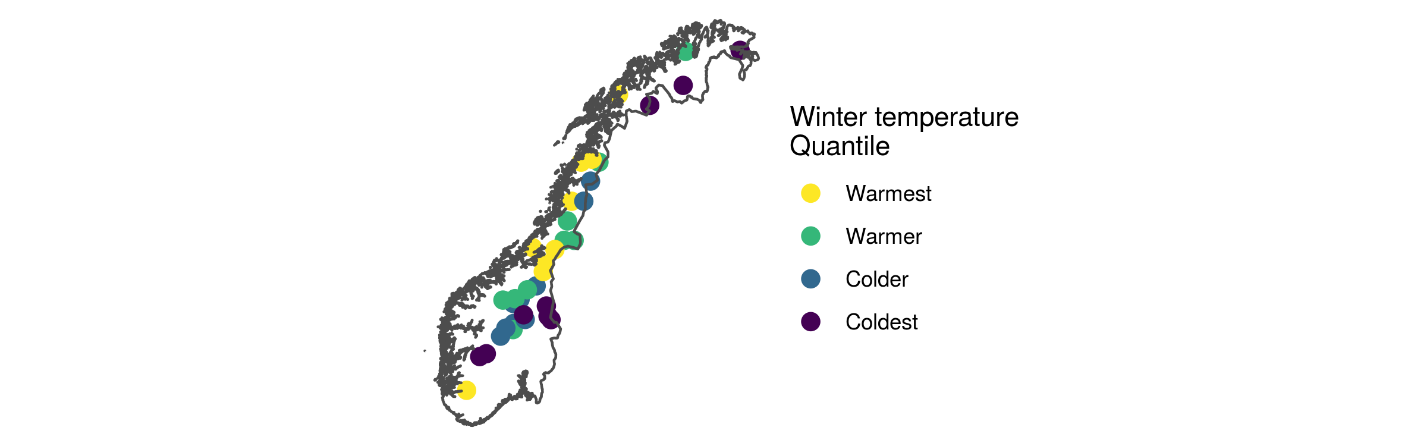


**Fig. S3** Location of the 36 survey regions, shaded by the winter temperature quartiles shown in Fig. 4


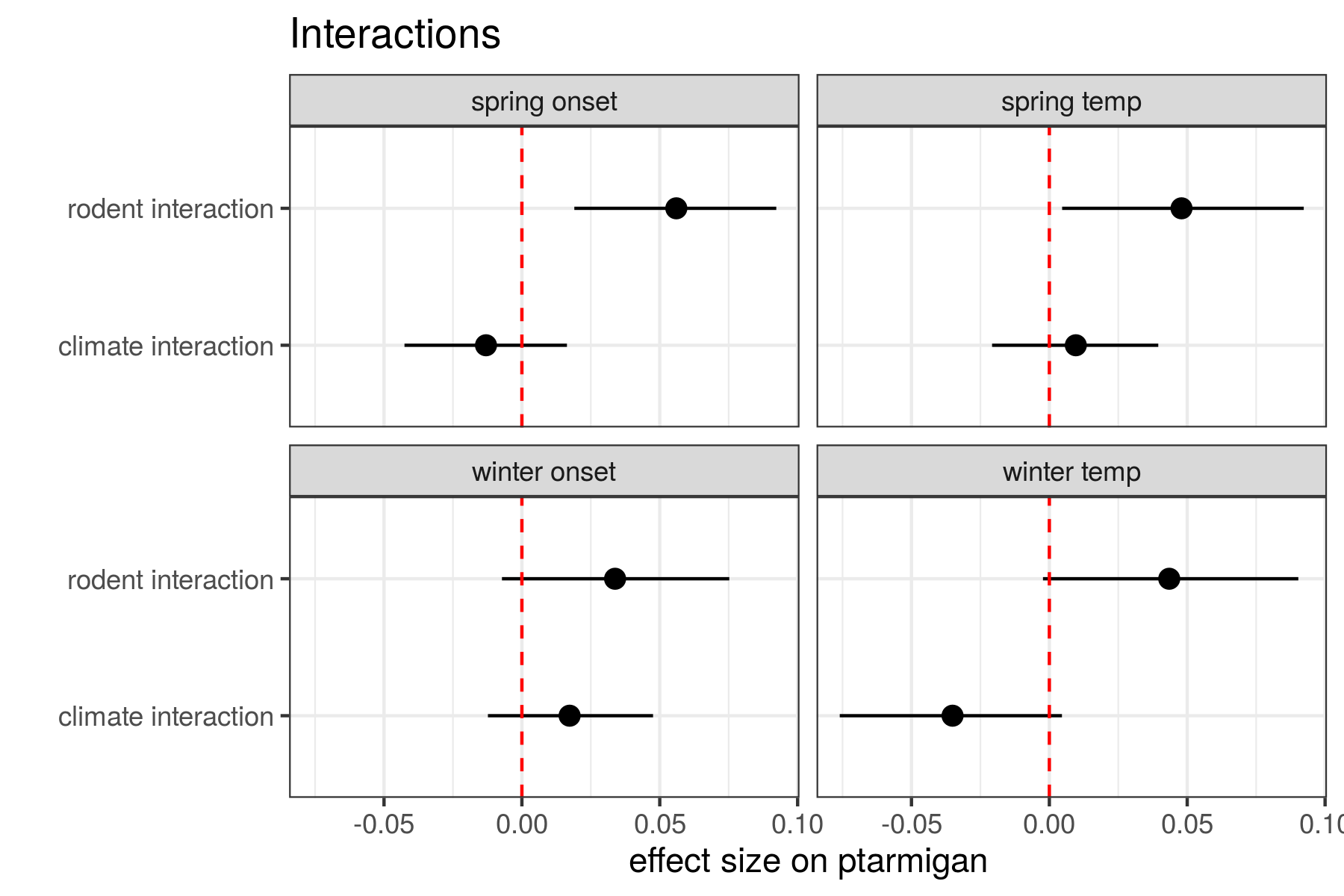


**Fig. S4** Coefficients for the interactive effects on ptarmigan of either between rodent occurrence and the spatial climatic variable or between temporal climatic anomalies and the spatial climatic variables. The points and lines are the means and 95% credible intervals. Positive effects reflect an increase in the effects of rodents or temporal climatic variation with increasing climatic harshness. In each model, the climatic variables is winter or spring temperature, or winter or spring onset.

**Table S1** Spatial and climatic variability of climate and rodent occurrence along the climatic gradient.

| Spatial gradient (°C) | | SD in Temporal climatic anomalies (°C) | | | | SD in rodent occurrence (proportion of occupied transects) | | | |
| --- | --- | --- | --- | --- | --- | --- | --- | --- | --- |
| Variable | SD | Coldest quartile | Colder  quartile | Warmer quartile | Warmest quartile | Coldest quartile | Colder  quartile | Warmer quartile | Warmest quartile |
| Spring  Temp | 1.343 | 1.227 | 1.251 | 1.234 | 1.156 | 0.395 | 0.380 | 0.339 | 0.226 |
| Winter  Temp | 2.044 | 2.633 | 2.385 | 2.382 | 2.270 | 0.318 | 0.412 | 0.339 | 0.229 |
| Spring  Onset | 12.429 | 15.535 | 11.983 | 10.324 | 11.184 | 0.358 | 0.412 | 0.313 | 0.302 |
| Winter  Onset | 8.529 | 10.447 | 12.540 | 12.916 | 14.891 | 0.395 | 0.336 | 0.326 | 0.300 |

Appendix A: Harvesting data

From the Statistics Norway website (www.ssb.no), we obtained local data on the number of harvested willow ptarmigan each hunting season. The hunting season in Norway usually opens September 10 (i.e. after the annual transect surveys) and ends on February 28 or March 15 in the following year. Harvest data were only available at the local administrative level of municipality (only 2008 onwards) and a larger-scale administrative level of county. In our analysis, we used the finer spatial resolution of municipality. Data for each hunting season were treated as coming from the year of the season start (i.e., the harvest for winter 2008/2009 was aligned to the year 2008). To impute the local harvest data for 2007, we used a simple linear regression model, using the 2008–2017 data to predict the log municipality harvest numbers from the log county harvest numbers. We used our model to predict the 2007 municipality data from the 2007 county data. The R2 of this model was 0.95, suggesting a good predictive ability for the imputation.

This variable was included in our hierarchical model of ptarmigan as described in the main text. The results (see figure below) showed that there was no detectible effect of harvesting. However, given the coarseness of the harvesting data, this is only a weak test of the effect of harvesting. Moreover, ptarmigan year-year change is largely driven by variation in recruitment, so effects of relative small variation in harvest pressure would be not be expected to show a strong signal.


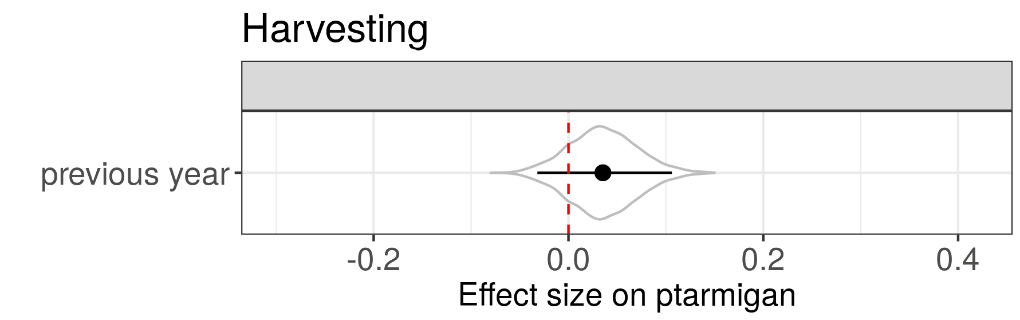


Figure for SOM A Effect of harvesting on changes in ptarmigan population density, as tested in the model presented in Fig. 4 of the main paper. Covariates were standardized to units of standard deviation to facilitate comparison of effect sizes. Violins represent the full posterior distributions. Point and ranges represent mean effects and 95% credible intervals of the distributions. The dashed vertical red line is the line of no effect.

Appendix B: Alternative rodent model

In the main text, we present a model in which rodent dynamics are modelled as a series of random effects for year (as a factor), survey region and year/survey region. This approach was chosen to minimise the assumptions made about the rodent dynamics and make testing their effects as comparable as possible to the climate data.

As an alternative to the random-effects rodent model, we also developed a rodent model based on an autoregressive model of ar2 – often used to model cyclic dynamics. Hence, rodent occurrence in year t was assumed to depend on rodent occurrences in year t-1 and t-2. Additionall, we allowed the strength of the ar1 and 2 processes to randomly vary among survey regions (see model code at the end). We included an additional random site-effect to smooth over remaining site variation.

The output of this model revealed similar cyclic dynamics to the predictions of the random-effects model, as shown shown. Moreover, this model revealed similar effects of rodents on the ptarmigan – with an increasingly positive effect of rodents (in the same year) with increasing climatic harshness, also shown below. Hence, in the main text, we retained the simpler random-effects model.


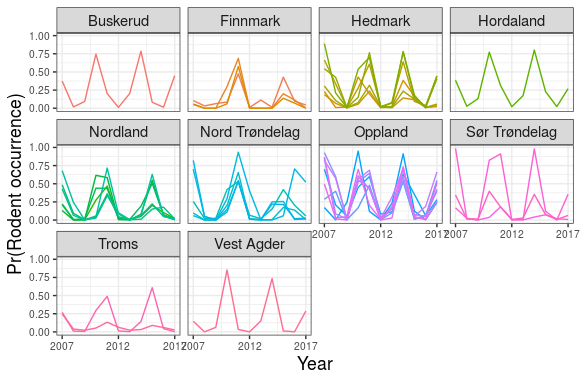


**Figure for SOM B** Predicted changes in rodent occurrence probability as predicted by an ar2 rodent model. The panels represent different counties in Norway. Each coloured line represents a survey region in our data and analysis.


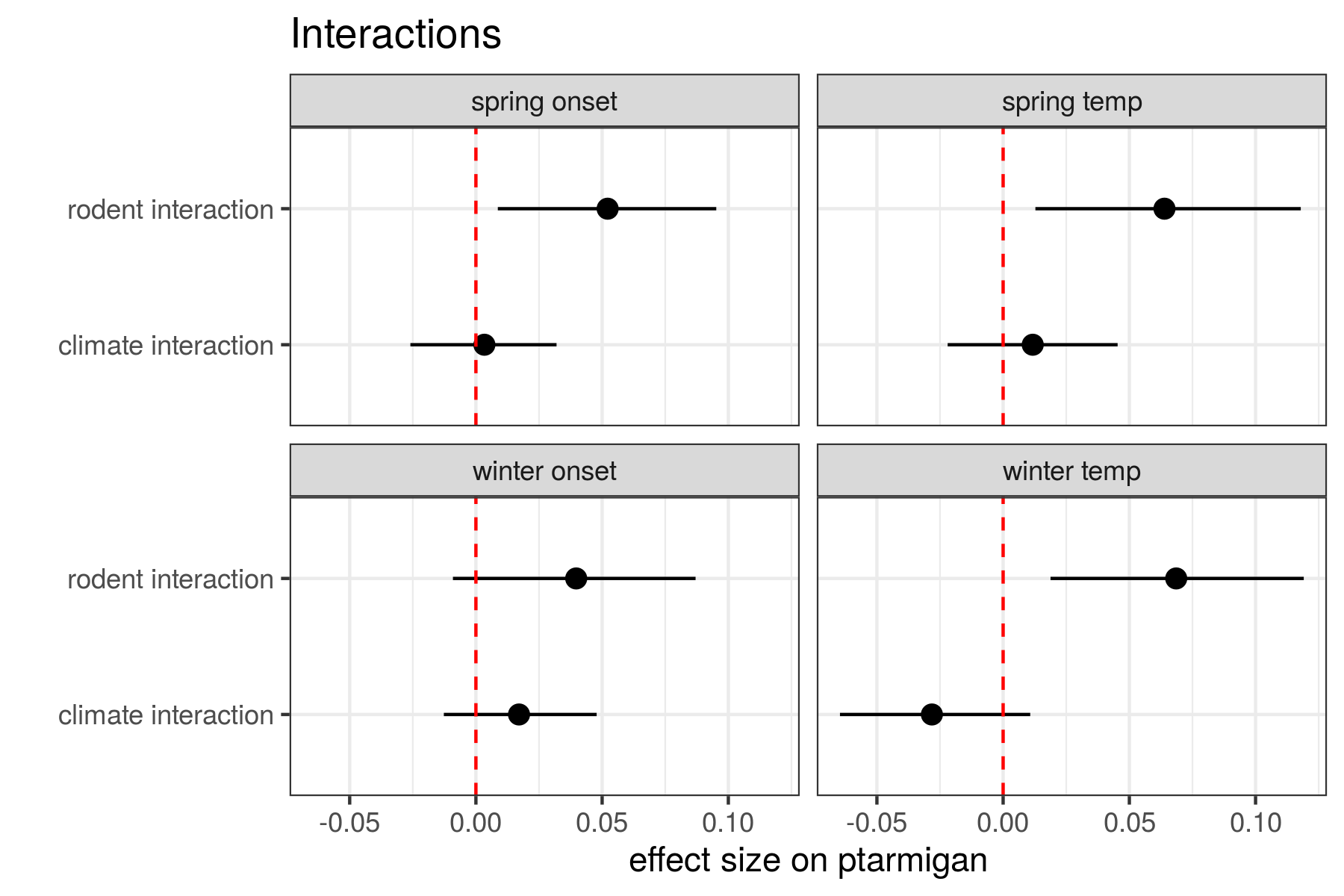


**Figure for SOM B** Coefficients for the interactive effects on ptarmigan of either between rodent occurrence and the spatial climatic variable or between temporal climatic anomalies and the spatial climatic variables, when the rodents as modelled as an ar2 process. The points and lines are the means and 95% credible intervals. Positive effects reflect an increase in the effects of rodents or temporal climatic variation with increasing climatic harshness. In each model, the climatic variables is winter or spring temperature, or winter or spring onset.

#################ar2 JAGS model for rodent data#########################################

#year 1 data

for(j in 1:n.Lines){

my.rodents[j,1] ~ dbern(rodent.year1[j])

logit(rodent.year1[j]) <- int.r + random.r.site[site[j]]

}

#year 2 data

for(j in 1:n.Lines){

my.rodents[j,2] ~ dbern(rodent.year2[j])

logit(rodent.year2[j]) <- int.r2 +

beta_ar0[site[j]] * logit(rodent.year1[j])+

random.r2.site[site[j]]

}

#model for subsequent years

for(j in 1:n.Lines){

rodent.p[j,1] <- rodent.year1[j]

rodent.p[j,2] <- rodent.year2[j]

for(t in 3:n.Years){

my.rodents[j,t] ~ dbern(rodent.p[j,t])

logit(rodent.p[j,t]) <- int.r3 +

beta_ar1[site[j]] * logit(rodent.p[j,t-1]) +

beta_ar2[site[j]] * logit(rodent.p[j,t-2]) +

random.r3.site[site[j]]

}

}

#priors

#intercepts for each model

int.r ~ dunif(-3,3)

int.r2 ~ dunif(-3,3)

int.r3 ~ dunif(-3,3)

#random slopes for each model

for(i in 1:n.Sites2){

beta_ar0[i] ~ dnorm(mu.ar0,tau.ar0)

beta_ar1[i] ~ dnorm(mu.ar1,tau.ar1)

beta_ar2[i] ~ dnorm(mu.ar2,tau.ar2)

}

mu.ar0 ~ dnorm(0,0.01)

mu.ar1 ~ dnorm(0,0.01)

mu.ar2 ~ dnorm(0,0.01)

sd.ar0 ~ dt(0, 1, 1)T(0,)

tau.ar0 <- pow(sd.ar0,-2)

sd.ar1 ~ dt(0, 1, 1)T(0,)

tau.ar1 <- pow(sd.ar1,-2)

sd.ar2 ~ dt(0, 1, 1)T(0,)

tau.ar2 <- pow(sd.ar2,-2)

#Random site effects

# year 1

site.r.sd ~ dt(0, 1, 1)T(0,)

site.r.tau <- pow(site.r.sd,-2)

for(j in 1:n.Sites2){

random.r.site[j] ~ dnorm(0,site.r.tau)

}

# year2

site.r2.sd ~ dt(0, 1, 1)T(0,)

site.r2.tau <- pow(site.r2.sd,-2)

for(j in 1:n.Sites2){

random.r2.site[j] ~ dnorm(0,site.r2.tau)

}

#other years

site.r3.sd ~ dt(0, 1, 1)T(0,)

site.r3.tau <- pow(site.r3.sd,-2)

for(j in 1:n.Sites2){

random.r3.site[j] ~ dnorm(0,site.r3.tau)

}

}

Appendix C: Bayesian hierarchical model

model{

#################

#Detection model#

#################

pi <- 3.141593

# priors for fixed effect parms for half-normal detection parm sigma

b.df.0 ~ dunif(0,20)

b.group.size ~ dnorm(0,0.1)

#random site2 effect (site2 = "survey region" throughout)

for(i in 1:n.Sites2){

random.df.site2[i] ~ dnorm(0,site2.df.tau)

}

site2.df.tau <- pow(site2.df.sd,-2)

site2.df.sd ~ dt(0, 1, 1)T(0,)

#random line effect

for(i in 1:n.Lines){

random.df.line[i] ~ dnorm(0,line.df.tau)

}

line.df.tau <- pow(line.df.sd,-2)

line.df.sd ~ dt(0, 1, 1)T(0,)

#model for variation in detectability

for(i in 1:N){

#linear predictor

mu.df[i] <- b.df.0 + b.group.size * detectionGroupSize[i] +

random.df.site2[detectionSite2[i]] +

random.df.line[detectionLine[i]]

# estimate of sd and var, given coeffs above

sig.df[i] <- exp(mu.df[i])

sig2.df[i] <- sig.df[i]*sig.df[i]

# effective strip width

esw[i] <- sqrt(pi * sig2.df[i] / 2)

f0[i] <- 1/esw[i] #assuming all detected on the line

# LIKELIHOOD

# using zeros trick

L.f0[i] <- exp(-y[i]*y[i] / (2*sig2.df[i])) * 1/esw[i] #y are the distances

nlogL.f0[i] <- -log(L.f0[i])

zeros.dist[i] ~ dpois(nlogL.f0[i])

}

#using this model and predicted group size (below), get predicted ESW for each line and year

for(t in 1:n.Years){

for(j in 1:n.Lines){

pred.sig[j,t] <- exp(b.df.0+

b.group.size * log(predGroupSize[j,t]+1)+

random.df.line[line[j]]+

random.df.site2[site2[j]])

pred.sig2[j,t] <- pow(pred.sig[j,t],2)

predESW[j,t] <- sqrt(pi * pred.sig2[j,t] / 2)

}

}

##################

#Group size model#

##################

#priors

int.gs ~ dnorm(0,0.01)

#random line effect

line.sd ~ dt(0, 1, 1)T(0,)

line.tau <- pow(line.sd,-2)

for(j in 1:n.Lines){

random.gs.line[j] ~ dnorm(0,line.tau)

}

#random site2 effect

site2.sd ~ dt(0, 1, 1)T(0,)

site2.tau <- pow(site2.sd,-2)

for(j in 1:n.Sites2){

random.gs.site2[j] ~ dnorm(0,site2.tau)

}

#random year effect

year.sd ~ dt(0, 1, 1)T(0,)

year.tau <- pow( year.sd,-2)

for(t in 1:n.Years){

random.gs.year[t] ~ dnorm(0, year.tau)

}

#random line/year effect

line.year.sd ~ dt(0, 1, 1)T(0,)

line.year.tau <- pow( line.year.sd,-2)

for(j in 1:n.Lines){

for(t in 1:n.Years){

random.gs.line.year[j,t] ~ dnorm(0, line.year.tau)

}

}

#random site2/year effect

site2.year.sd ~ dt(0, 1, 1)T(0,)

site2.year.tau <- pow(site2.year.sd,-2)

for(j in 1:n.Sites2){

for(t in 1:n.Years){

random.gs.site2.year[j,t] ~ dnorm(0, site2.year.tau)

}

}

#Model

#for each detection, model group size

for(i in 1:N){

GroupSize[i] ~ dpois(expGroupSize[i])

log(expGroupSize[i]) <- int.gs + random.gs.year[detectionYear[i]] +

random.gs.line[detectionLine[i]] +

random.gs.site2[detectionSite2[i]]+

random.gs.line.year[detectionLine[i],detectionYear[i]] +

random.gs.site2.year[detectionSite2[i],detectionYear[i]]

}

#using this model, get predicted group size for each line and year

for(t in 1:n.Years){

for(j in 1:n.Lines){

log(predGroupSize[j,t]) <- int.gs + random.gs.year[t] +

random.gs.line[j] +

random.gs.site2[site2[j]]+

random.gs.line.year[j,t] +

random.gs.site2.year[site2[j],t]

}

}

###########################

#Model of ptarmigan density

###########################

#priors

#intercept

int.d ~ dnorm(0,0.001)

#random line effect

line.d.sd ~ dt(0, 1, 1)T(0,)

line.d.tau <- pow(line.d.sd,-2)

for(j in 1:n.Lines){

random.d.line[j] ~ dnorm(0,line.d.tau)

}

#random site2 effect

site2.d.sd ~ dt(0, 1, 1)T(0,)

site2.d.tau <- pow(site2.d.sd,-2)

for(j in 1:n.Sites2){

random.d.site2[j] ~ dnorm(0,site2.d.tau)

}

#random year

year.d.sd ~ dt(0, 1, 1)T(0,)

year.d.tau <- pow(year.d.sd,-2)

for(t in 1:(n.Years-1)){

random.d.year[t] ~ dnorm(0,year.d.tau)

}

#priors for density in year 1 only

int.d1 ~ dnorm(0,0.001)

#line effect - year 1

line1.d.sd ~ dt(0, 1, 1)T(0,)

line1.d.tau <- pow(line1.d.sd,-2)

for(j in 1:n.Lines){

random.d.line1[j] ~ dnorm(0,line1.d.tau)

}

#slopes for fixed effects

beta.auto ~ dunif(-1.5,-0.01)

beta_harvest ~ dnorm(0,0.001)

beta.covariateS ~ dnorm(0,0.001)

beta.covariateT ~ dnorm(0,0.001)

beta.covariate_rodT ~ dnorm(0,0.001)

beta.covariate_rodTL ~ dnorm(0,0.001)

#interaction

beta.covariate_intC ~ dnorm(0,0.001)

beta.covariate_intR ~ dnorm(0,0.001)

#Dispersion parameter

r ~ dunif(0.001,50)

#Model for ptarmigan density - link to detection model

for(j in 1:n.Lines){

for(t in 1:n.Years){

NuIndivs[j,t] ~ dnegbin(p[j,t],r)

p[j,t] <- r/(r+Nu[j,t])

Nu[j,t] <- Density[j,t] * TransectLength[j,t]/1000 * (predESW[j,t]/1000) *2

Density[j,t] <- exp(x[j,t])

}

}

#model for density in year one

for(j in 1:n.Lines){

x[j,1] <- int.d1 + random.d.line1[j]

}

#model for density in subsequent years

for(j in 1:n.Lines){

for(t in 2:n.Years){

x[j,t] <- int.d + x[j,t-1] +

beta.auto * (x[j,t-1]-1.7) +

beta_harvest * log_bkh[j,t-1] +

random.d.line[j] +

random.d.site2[site2[j]]+

random.d.year[t-1]+

beta.covariateS * spatialMatrix1[j] +

beta.covariateT * temporalMatrix1[j,t] +

beta.covariate_rodT * rodent.p[j,t] +

beta.covariate_rodTL * rodent.p[j,t-1] +

beta.covariate_intC * spatialMatrix1[j] * temporalMatrix1[j,t]+

beta.covariate_intR * spatialMatrix1[j] * rodent.p[j,t]

}

}

#predicted change in effects of climate and rodents along the climatic gradient

#climatic gradient is simply a 1000 length sequence of values between min and min of the spatial climate variable

for(i in 1:n.Preds){

predsC[i] <- beta.covariateT + beta.covariate_intC * climaticGradient[i]

predsR[i] <- beta.covariate_rodT + beta.covariate_intR * climaticGradient[i]

}

#sub model for rodent data:

for(j in 1:n.Lines){

for(t in 1:n.Years){

my.rodents[j,t] ~ dbern(rodent.p[j,t])

logit(rodent.p[j,t]) <- random.r.year[t]+

random.r.site2[site2[j]]+

random.r.site2.year[site2[j],t]

}

}

#priors

#intercept

mu.r ~ dunif(0.0001,0.999)

int.r <- logit(mu.r)

#random year effect

year.r.sd ~ dt(0, 1, 1)T(0,)

year.r.tau <- pow(year.r.sd,-2)

for(t in 1:n.Years){

random.r.year[t] ~ dnorm(int.r,year.r.tau)

}

#random site2 effect

site2.r.sd ~ dt(0, 1, 1)T(0,)

site2.r.tau <- pow(site2.r.sd,-2)

for(j in 1:n.Sites2){

random.r.site2[j] ~ dnorm(0,site2.r.tau)

}

#random site2/year effect

site2.year.r.sd ~ dt(0, 1, 1)T(0,)

site2.year.r.tau <- pow(site2.year.r.sd,-2)

for(j in 1:n.Sites2){

for(t in 1:n.Years){

random.r.site2.year[j,t] ~ dnorm(0,site2.year.r.tau)

}

}

#simulate fitted values from the ptarmigan model

for(j in 1:n.Lines){

for(t in 1:n.Years){

Nu.new[j,t] ~ dnegbin(p[j,t],r)

}

}

}
